# Successful Spinal Cord Stimulation for Necrotizing Raynaud’s Phenomenon in COVID-19 Affected Patient: The Nightmare Comes Back

**DOI:** 10.7759/cureus.14569

**Published:** 2021-04-19

**Authors:** Mariateresa Giglio, Angela Preziosa, Martina Rekatsina, Omar Viswanath, Ivan Urits, Giustino Varrassi, Antonella Paladini, Filomena Puntillo

**Affiliations:** 1 Department of Anaesthesiology, University of Bari Aldo Moro, Bari, ITA; 2 Department of Anesthesiology, University of Bari Aldo Moro, Bari, ITA; 3 Pain Management, Whipps Cross Hospital Barts Health National Health Service (NHS), London, GBR; 4 Department of Anesthesiology, University of Arizona, Phoenix, USA; 5 Department of Anesthesia, Critical Care and Pain Medicine, Beth Israel Deaconess Medical Center, Harvard Medical School, Boston, USA; 6 Department of Research and Development, Paolo Procacci Foundation, Roma, ITA; 7 Dipartimento di Medicina clinica, sanità pubblica, scienze della vita e dell'ambiente, University of L'Aquila, L'Aquila, ITA; 8 Department of Intedisciplinary Medicine, University of Bari Aldo Moro, Bari, ITA

**Keywords:** vascular pain, raynaud phenomenon, spinal cord stimulation, covid-19, pain

## Abstract

Necrotizing Raynaud’s phenomenon is a vascular clinical syndrome characterized by vasospasm of distal resistance vessels, usually triggered by cold temperatures or by psychological conditions such as anxiety and stress. Pain is the first reported symptom, related to insufficient oxygen delivery to the extremities that leads to ischemia of the peripheral tissues. The initial treatment is conservative, but if the symptoms persist, necrosis and distal amputation can occur. In selected patients, neuromodulation with spinal cord stimulation (SCS) can be an effective treatment by reducing pain and amputation rate. Recent evidence suggests that severe acute respiratory syndrome coronavirus 2 (SARS-CoV-2) can cause endotheliopathy with microvascular and macrovascular thrombotic events and can present as a systemic inflammatory vascular disease.

We present a case of a severe necrotizing Raynaud’s phenomenon successfully treated and controlled with SCS that abruptly reappeared during SARS-CoV-2 infection.

The report of this case is suggestive for potential treatment in case of peripheral ischemia consequent to COVID-19 vasculopathy. The interaction between SCS and SARS-CoV-2-related endotheliopathy is unknown and would deserve further studies.

## Introduction

Raynaud’s phenomenon (RP) is a pathological condition caused by an exaggerated cold-induced vasoconstriction. The typical manifestation is an extreme whiteness of the extremities, later leading to blue discoloration, and ultimately to ulcers [1]. Patients usually complain of severe pain due to ischemia, and the initial treatment consists of pharmacological therapies. In secondary RP, the ischemic attacks are more frequent and painful than in the primary one; asymmetric and digital ulcerations and necrosis can often occur. In selected cases, a trial of spinal cord stimulation (SCS) can successfully promote ulcer healing, pain control and prevent amputation [2-4].

The world’s medical community is continuing its battle with the outbreak of severe acute respiratory syndrome coronavirus 2 (SARS-CoV-2 or COVID-19). The common clinical presentations of the disease caused by COVID-19 are fever (98%), cough (76%), myalgia and fatigue (18% each), with accompanying leucopenia (25%) and lymphopenia (63%) [5-7]. Although diffuse alveolar damage and acute respiratory failure are the main features of COVID-19, evidence is emerging that the involvement of other organs and tissues are of pivotal importance [8]. Ongoing reports describe the hyper-coagulability and thrombotic tendency in COVID-19, as well as the damage to endothelium and the activation of inflammation leading to vasculitis [9,10]. The key pathogenetic mechanisms of the complex thrombo-inflammatory process induced by SARS-COV-2 seem to be the direct endothelial damage and the indirect damage caused by inflammation. Moreover, the increased activity of clotting factors, the loss of glycocalyx protective function and decreased nitric oxide levels may also contribute to coagulopathy, inflammation and thrombosis observed in COVID-19 [9]. In this scenario, the effects of COVID-19 on patients affected by RP are still not clarified.

In this case report, we describe a patient with a severe necrotizing RP involving both feet successfully controlled with SCS for almost four years [11], but this controlled clinical presentation abruptly reappeared during a SARS-CoV-2 infection.

## Case presentation

**Figure 1:**
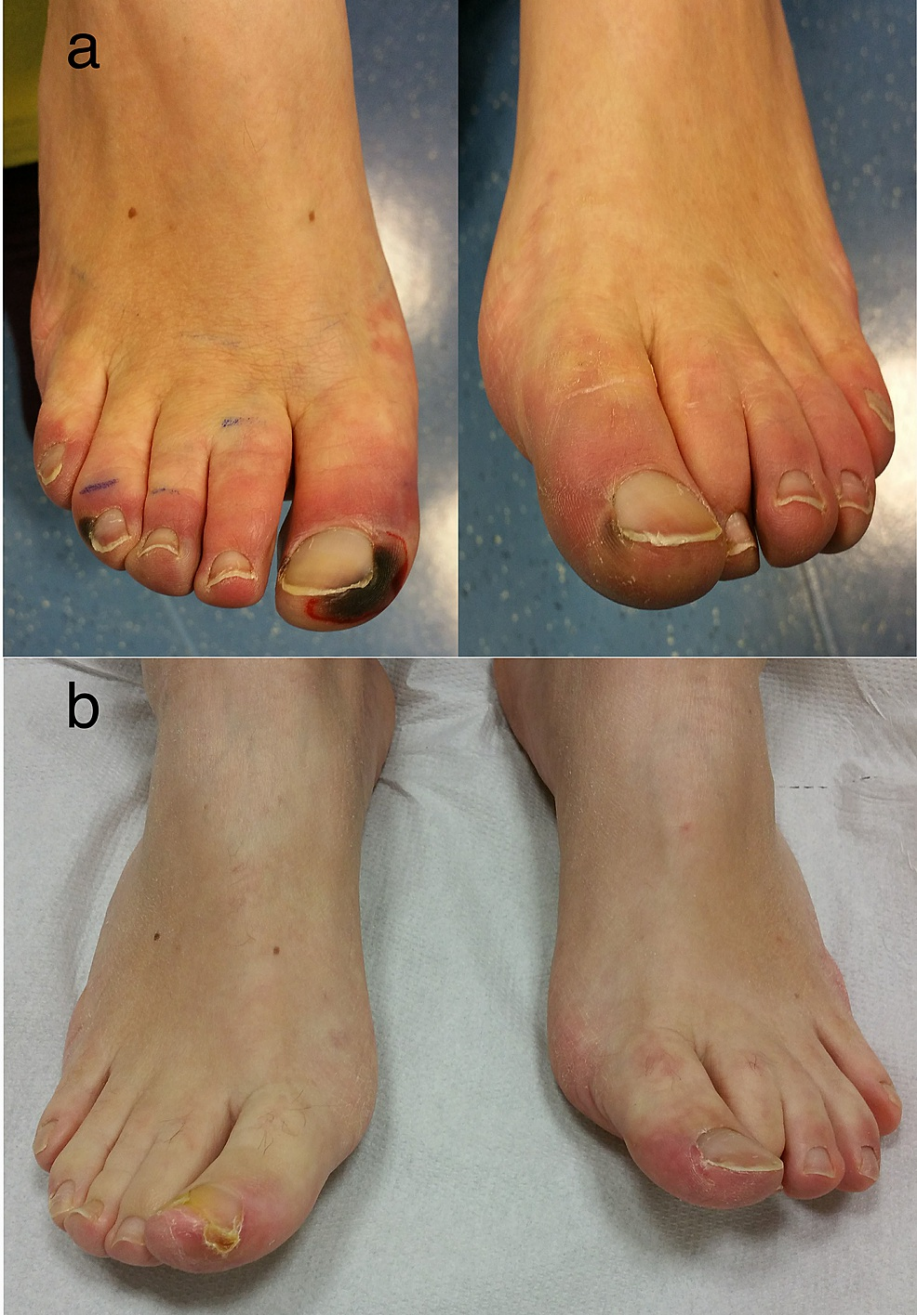
(a) Signs of incoming ischemia and necrotic ulcers of toes at admission in 2016, when diagnosis of necrotizing Raynaud’s phenomenon was made. (b) Complete remission of ulcers at two months, after spinal cord stimulator implant

**Figure 2:**
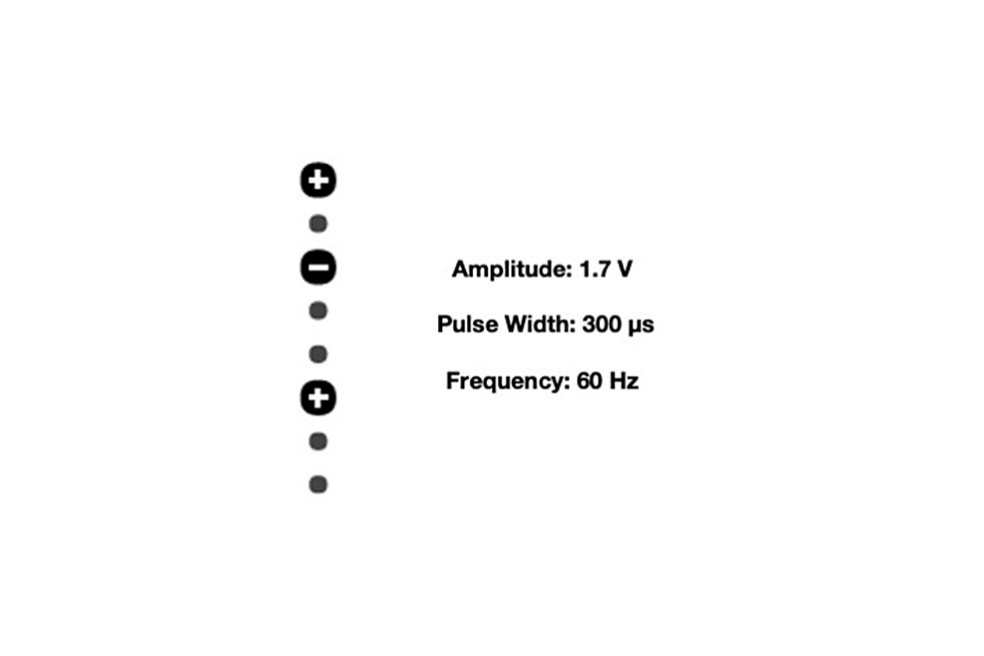
Stimulation parameters at implantation

The patient was a 37-year-old woman who in November 2016 had been diagnosed a necrotizing RP at both feet. She reported severe pain (visual analogue scale 9/10) involving both feet with sign of severe ischemia and necrotic ulcers of toes (Figure 1-a). The pain was described as distressing and hyperalgesic; allodynia including light touches increased the pain and any targeted pharmacological treatment, including vasodilator infusion and opioids failed to control it. The vasospastic attacks worsened with presentation of cold temperatures. A multimodal pain therapy was immediately started with gabapentin up to 900 mg/die and oral morphine 120 mg/die; moreover, intravenous iloprost (up to 2ng/kg/min for 24 hr for seven days) was started, but her pain and digital necrosis got worse. A trial of SCS was therefore proposed. One octopolar electrode (Vectris™ SureScan® MRI 1x8, Medtronic, Minneapolis, MN, USA) was positioned midline at T9-T10 and the stimulation was immediately started (stimulation parameters are showed in Figure 2). The day after, pain was described as 3/10 and perfusion in her feet improved significantly in the first postoperative days, and with it the risk of distal amputation quickly dissipated. Pain medication was gradually tapered down and eventually stopped, and her digit ulcers completely healed in two months (Figure 1-b). A diagnosis of undifferentiated connective tissue disease was subsequently made. The patient was continued to be seen through the outpatient clinic, with continued vasodilator infusion monthly and ongoing reporting of no pain.

**Figure 3:**
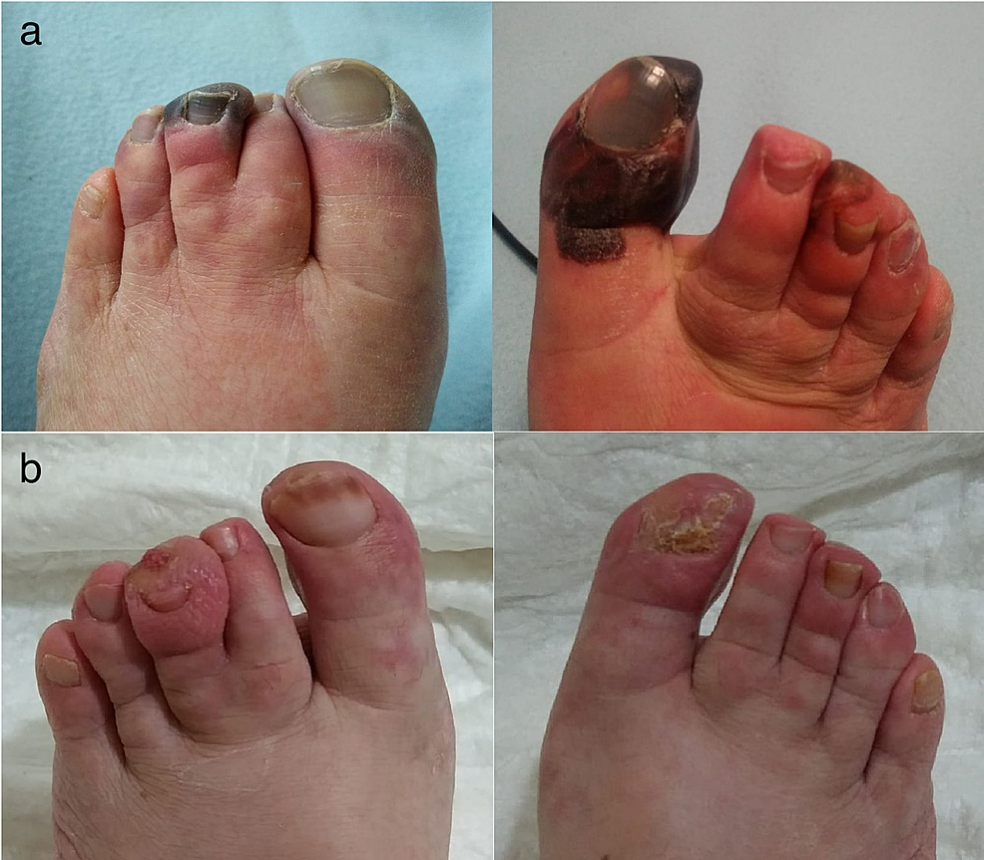
(a) Signs of incoming ischemia and necrotic ulcers in both feet after the admission in October 2020, at the admission in hospital. (b) Necrotic ulcers healing in January 2021

In October 2020, she was admitted again complaining of a sudden and severe increase in pain involving again both feet. Ischemic changes were evident at the extremities, with ulcers involving the toe and the third finger of both feet (Figure 3a). She reported that increasing the amplitude of her SCS did not lead to any improvement. The pain worsened in both supine and resting position, while only walking seemed to alleviate it. No other symptoms were described. A chest radiogram showed the correct position of the two electrodes, but also interstitial pneumonia. The pharyngeal swab for SARS-CoV-2 was undertaken, showing a positive result.

She was therefore admitted to the COVID-19 medical area of the Bari (Italy) University Hospital, and pain treatment was started, with gabapentin and oral morphine, that was gradually increased to 900 mg/die and 30 mg/die, respectively. SCS was reprogramed in order to obtain better coverage of paresthesia in the painful area and the amplitude of thoracic lead was incremented up to the tolerated level (5 Volts). In the subsequent days, the patient presented fever up to 39°C, with cough and dyspnea. Low flow (4 l/min) oxygen therapy was initiated with intravenous dexamethasone 6 mg/day. A combined therapy with subcutaneous low weight heparin (enoxaparin 4,000 UI/day), aspirin (100 mg/day) and iloprost infusion (up to 2 ng/kg/min for 24 hr for seven days) was also started in order to improve peripheral perfusion. After 10 days, respiratory symptoms improved so that she did not need additional supplemental oxygen. Severe pain at both feet was reported in the following month, and tissue discoloration persisted. The patient was tested again with a negative result for SARS-CoV-2 after 30 days. Only after 60 days after symptoms started, tissue perfusion improved (Figure 3b), the pain was successfully controlled, anticonvulsants and opioids were gradually reduced and then stopped. The amplitude of SCS was then also reduced.

## Discussion

There are multiple sources of evidence that show the beneficial effect of SCS in treating RP, both in terms of pain reduction and better perfusion [3,4], even if the mechanism is not fully understood yet [12]. One hypothesis is that SCS activates low-threshold, large-diameter A-beta fibers, which synapse onto inhibitory (GABA-ergic or cholinergic) interneurons in the spinal dorsal horn. These interneurons release inhibitory neurotransmitters (e.g., GABA), thus reducing the excitability of spinal second-order neurons and attenuating stimulus by A-delta and C-fibers [3,4]. Others proposed that pain relief can be mediated by suppression of nociceptive transmission via descending inhibitory pathways [13]. Moreover, the antidromic activation of afferent fibers in the dorsal roots causes the peripheral release of calcitonin gene-related peptide (CGRP). This would enable cutaneous vasodilation [14] by means of nitric oxide release by endothelial cells, thus increasing peripheral blood flow. In addition, SCS induces inhibition of efferent vasoconstrictor sympathetic activity, transmitted via nicotinic receptors in the ganglia and mainly via alpha_1_ receptors on the vessels [2,4], thereby increasing blood flow.

The vascular pathology of COVID-19 is a topic of great interest. Macro and microvascular thrombosis involving arteries, veins, arterioles, capillaries and venules in all major organs were reported [15]. The virus elicits an inflammatory response with macrophage activation and increased cytokines levels (the so-called “cytokine storm”) [16] with associated hyper-coagulability, characterized by elevated D-dimer, fibrinogen, factor VIII and von Willebrand factor. This may be worsened during conditions of oxidative stress [17]. The activation of macrophage and neutrophils, together with the increased release of cytokines and chemokines such as TNF-α, IL-1 and IL-8, can contribute to endothelial injury, promoting an inflammatory, prothrombotic state [18]. Moreover, the virus directly infects the vascular endothelial cell by binding to angiotensin-converting enzyme 2 (ACE-2) [19] and the transmembrane protease serine 2 (TMPRSS-2), causing cellular damage and apoptosis, thus favoring clot formation. In addition, infected endothelial cells lose their ability to regulate vascular tone, since the production of nitric oxide by nitric oxide synthase is impaired. This impairment causes, in turn, an increase of leukocyte and platelet adhesion, stimulates inflammatory cell migration into the vessel wall and smooth muscle cell proliferation, favoring apoptosis, inflammation and vasoconstriction [9]. Finally, cytokines mediate the activation of proteases that partially disrupt the glycocalyx layer, further increasing leukocyte recruitment and extravasation [20].

In this context, the effects of SARS-COV-2 infection on patients affected by RP are still under study. Even if neuromodulation, in general, seems beneficial in many pain syndromes [21], the interplay between the action of SCS in restoring perfusion on one side and the pro-inflammatory and procoagulant effect on micro and macrovasculature of the virus on the other is largely unknown.

## Conclusions

This is the first report, to our knowledge, of a case of a severe necrotizing RP successfully treated with SCS, which suddenly got worse after SARS-CoV-2 infection. The viral load seemed to activate vasoconstriction and impair tissue perfusion, after four years of excellent pain and tissue perfusion control obtained with SCS. We may not be sure whether the neuromodulation of the SCS may have contributed to pain control during the viral infection and to tissue healing, and this is a clear limit of the study. Certainly, the hypothesis is very suggestive.

Further trials are needed to elucidate the interaction between RP and the effects of vascular dysfunction in COVID-19. Moreover, the role of SCS in this particular setting may be an intriguing topic for incoming studies.
